# Dexmedetomidine and clonidine in epidural anaesthesia: A comparative evaluation

**DOI:** 10.4103/0019-5049.79883

**Published:** 2011

**Authors:** Sukhminder Jit Singh Bajwa, Sukhwinder Kaur Bajwa, Jasbir Kaur, Gurpreet Singh, Vikramjit Arora, Sachin Gupta, Ashish Kulshrestha, Amarjit Singh, SS Parmar, Anita Singh, SPS Goraya

**Affiliations:** Department of Anaesthesiology and Intensive Care, Gian Sagar Medical College & Hospital, Banur, Punjab, India; 1 Department of Obstetrics & Gynaecology, Gian Sagar Medical College & Hospital, Banur, Punjab, India

**Keywords:** Clonidine, dexmedetomidine, epidural anaesthesia, ropivacaine, vaginal hysterectomy

## Abstract

Efforts to find a better adjuvant in regional anaesthesia are underway since long. Aims and objectives are to compare the efficacy and clinical profile of two α-2 adrenergic agonists, dexmedetomidine and clonidine, in epidural anaesthesia with special emphasis on their sedative properties and an ability to provide smooth intra-operative and post-operative analgesia. A prospective randomized study was carried out which included 50 adult female patients between the ages of 44 and 65 years of (American Society of Anaesthesiologists) ASAI/II grade who underwent vaginal hysterectomies. The patients were randomly allocated into two groups; ropivacaine + dexmedetomidine (RD) and ropivacaine + clonidine (RC), comprising of 25 patients each. Group RD was administered 17 ml of 0.75% epidural ropivacaine and 1.5 μg/kg of dexmedetomidine, while group RC received admixture of 17 ml of 0.75% ropivacaine and 2 μg/kg of clonidine. Onset of analgesia, sensory and motor block levels, sedation, duration of analgesia and side effects were observed. The data obtained was subjected to statistical computation with analysis of variance and chi-square test using statistical package for social science (SPSS) version 10.0 for windows and value of *P* < 0.05 was considered significant and *P* < 0.0001 as highly significant. The demographic profile, initial and post-operative block characteristics and cardio-respiratory parameters were comparable and statistically non-significant in both the groups. However, sedation scores with dexmedetomidine were better than clonidine and turned out to be statistically significant (*P* < 0.05). The side effect profile was also comparable with a little higher incidence of nausea and dry mouth in both the groups which was again a non-significant entity (*P* > 0.05). Dexmedetomidine is a better neuraxial adjuvant compared to clonidine for providing early onset of sensory analgesia, adequate sedation and a prolonged post-operative analgesia.

## INTRODUCTION

Surgical methods and the anaesthetic techniques have evolved and improved drastically over the last two decades. Many techniques and drug regimens, with partial or greater success, have been tried from time to time to calm the patients and to eliminate theanxiety component during regional anaesthesia.[1–3] The fear of surgery, the strange surroundings of the operation theatre, the sight and sound of sophisticated equipment, dynamicity of an ‘operation’ during regional anaesthesia and the masked faces of so many strange personale makes the patient panic to any extent.[4,5] The intense sensory and motor block, continuous supine position for a prolonged duration and the inability to move the body during regional anaesthesia brings a feeling of discomfort and phobia in many of the patients.[6] The high cephalic spread of analgesia with local anaesthetics may be significant but still its quality sometimes may not correlate with the level of sensory analgesia.[7] At this stage, the impulsive use of large doses of sedation or even general anaesthesia with mask defeats the novel purpose of regional anaesthesia whereby a continuous verbal contact with the patient is lost. Sedation, stable haemodynamics and an ability to provide smooth and prolonged post-operative analgesia are the main desirable qualities of an adjuvant in neuraxial anaesthesia.

α-2 adrenergic agonists have both analgesic and sedative properties when used as an adjuvant in regional anaesthesia.[8–13] Dexmedetomidine is a highly selective α_2_Adrenergic agonist with an affinity of eight times greater than clonidine. There is no such study which has compared the dose equivalence of these drugs but the observations of various studies have stated that the dose of clonidine is 1.5–2 times higher than dexmedetomidine when used in epidural route.[14–19] The anaesthetic and the analgesic requirement get reduced to a huge extent by the use of these two adjuvants because of their analgesic properties and augmentation of local anaesthetic effects as they cause hyperpolarisation of nerve issues by altering transmembrane potential and ion conductance at locus coeruleus in the brainstem.[20–24] The stable haemodynamics and the decreased oxygen demand due to enhanced sympathoadrenal stability make them very useful pharmacologic agents.[25,26]

Keeping their pharmacologic interactions and other properties we planned a double blind prospective randomized clinically controlled study at our institute with an aim to compare the analgesic and sedative effects of both these drugs when used epidurally as an adjuvant to ropivacaine in patients undergoing vaginal hysterectomy.

## METHODS

After obtaining the permission of appropriate authority of the institute and written consent from patients, 50 female patients of ASA (American Society of Anaesthesiologists) grade I and II between the ages of 44 and 65 years were enrolled for the study that underwent vaginal hysterectomies. The patients with haematological disease, bleeding or coagulation test abnormalities, psychiatric diseases, diabetes, history of drug abuse and allergy to local anaesthetics of the amide type were excluded from the study.

Patients were randomly allocated to one of the following two treatment groups in a double blinded fashion based on a computer-generated code: Ropivacaine + clonidine (RC), Ropivacaine + dexmedetomidine (RD) and were administered tablet Ranitidine 150 mg as premedicant a night before and on the morning of the surgery. In the operation theatre, a good intravenous access was secured and monitoring devices were attached which included heart rate, electrocardiograph (ECG), pulse oximetry (SpO_2_), non-invasive blood pressure (NIBP), respiratory rate and the baseline parameters were recorded. The drug syringes were prepared by an anaesthesia technician who was unaware of the proceedings. Patients were administered epidural block with 18 gauge Touhy needle and catheter was secured 3–4 cm into epidural space and a test dose of 3 ml of 2% lignocaine hydrochloride solution containing adrenaline 1:200,000 was injected. After 4–6 minutes of administering the test dose, patients in group RC received 17 ml of 0.75% ropivacaine and 2 μg/kg of clonidine. Patients in group RD were administered 17 ml solution of 0.75% ropivacaine and 1.5 μg/kg of dexmedetomidine. The bilateral pin-prick method was used to evaluate and check the sensory level while a modified Bromage scale (0 = no block, 1 = inability to raise extended leg, 2 = inability to flex knee and 3 = inability to flex ankle and foot) was used to measure the motor blockade effect at 5, 10, 15, 20, 25 and 30 minutes intervals after the epidural administration of the drugs. The surgical position was made approximately after 25–30 minutes of epidural administration of drugs in every patient after complete establishment of sensory and motor block. The following block characteristics were observed and recorded: initial period of onset of analgesia; the highest dermatomal level of sensory analgesia; the complete establishment of motor blockade, the time to two segment regression of analgesic level, regression of analgesic level to S1 dermatome and time to complete recovery. Grading of sedation was evaluated by a five-point scale (1-alert and wide awake, 2-arousable to verbal command, 3-arousable with gentle tactile stimulation, 4-arousable with vigorous shaking and 5-unarousable). Sedation scores were recorded just before the initiation of surgery and thereafter every 20 minutes during the surgical procedure.

Cardio-respiratory parameters were monitored continuously and recordings were made every 5 min until 30 min and at 10 min interval, thereafter up to 60 min and then at 15 min interval for next hour and finally at 30 min in the third hour. Hypotension (defined as systolic arterial pressure falling more than 20% mmHg) was treated with inj. mephenteramine 3–6 mg in bolus doses and heart rate <50 beats/min was treated with 0.3 mg of inj. atropine. Intravenous fluids were given as per body weight and operative loss requirement. During the surgical procedure, adverse event like anxiety, nausea, vomiting, pruritis, shivering, etc. were recorded. Nausea and vomiting were treated with 6 mg of intravenous ondansetron.

All the vital and haemodynamic parameters were recorded in the recovery room also at 1, 5, 10, 20 and 30 min interval. The onset of pain was managed by top-up doses of 8 ml of 0.2% ropivacaine after operation. At the end of study, all the data was compiled systematically and analyzed using ‘Analysis of variance and chi-square test. Statistical package for social science (SPSS) version 10.0 for windows, Chicago III was used to compare the continuous variables between the two groups. Value of *P* < 0.05 was considered significant and *P* < 0.0001 as highly significant.

## RESULTS

A total of 50 patients who underwent vaginal hysterectomy were enrolled for the study and were randomly divided into two groups. The demographic profiles of the patients in both the groups were comparable with regards to age, weight and body mass index. The distribution as per ASA status was similar in both the groups and mean duration of surgery was comparable in both the groups and statistically non significant (*P* > 0.05) [Table 1].

**Table 1:** The demographic profile of patients of both the groups

Demographic characteristics	RD (n = 25)	RD (n = 25)	*P*
Age (years)	50.38 ± 8.64	52.06 ± 6.36	0.66
Weight (kg)	56.84 ± 10.52	58.26 ± 6.74	0.72
Body mass index	28.04 ± 3.46	29.32 ± 3.08	0.92
ASA (I/II)	21/4	20/5	0.81
Mean duration of surgery (min)	96.34 ± 14.58	99.78 ± 13.68	0.26

ASA - American Society of Anaesthesiologists, RC - Ropivacaine + clonidine, RD - Ropivacaine + dexmedetomidine

Addition of dexmedetomidine to ropivacaine as an adjuvant resulted in an earlier onset (8.52 ± 2.36 min) of sensory analgesia at T10 as compared to the addition of clonidine (9.72 ± 3.44 min). Dexmedetomidine not only provided a higher dermatomal spread but also helped in achieving the maximum sensory anaesthetic level in a shorter period (13.14 ± 3.96 min) compared to clonidine (15.80 ± 4.86 min). Modified Bromage scale 3 was achieved earlier (17.24 ± 5.16 min) in patients who were administered dexmedetomidine as adjuvant. All these initial block characteristics turned out to be statistically significant values on comparison (*P* < 0.05) [Table 2].

**Table 2:** Comparison of initial block characteristics in both the groups

Initial block characteristics	Group RD (n = 25)	Group RC (n = 25)	*P*
Onset time of sensory block at T10 (in minutes)	8.52 ± 2.36	9.72 ± 3.44	0.032
Maximum sensory block level	T5-6	T6-7	-
Time to maximum sensory block level (in minutes)	13.14 ± 3.96	15.80 ± 4.86	0.018
Time in minutes for complete motor block	17.24 ± 5.16	19.52 ± 4.06	0.041
Mean total dose of Mephenteramine requirement (mg)	10.6	8.4	0.76

*P<0.05-S, RC - Ropivacaine + clonidine, RD - Ropivacaine + dexmedetomidine

Dexmedetomidine is a popular sedative agent nowadays and similar findings were observed in our study as well. Mean sedation scores were significantly higher in RD group compared to RC group as 36% patients in group RD had a sedation score of 3 as compared 16% in group RC (*P* < 0.0001). Only 16% of the patients in the RD group had sedation scores of 1 compared to 32% wide and awake patients in RC group, which was a highly significant statistical entity (*P* < 0.0001) Table 3.

**Table 3:** Comparison of intra-operative sedation scores in patients of group RD and group RC

Sedation scores during surgery	Group RD No. of patients/ (%)	Group RC No. of patients/ (%)	*P*
1	4 (16)	8** (32)	<0.0001
2	12 (48)	13 (52)	0.65
3	9** (36)	4 (16)	<0.0001
4	0	0	-
5	0	0	-

** *P*<0.0001-HS, RC - Ropivacaine + clonidine, RD - Ropivacaine + dexmedetomidine

The findings of Table 4 reveal statistically significant values on comparison of post-operative block characteristics among the two groups. Dexmedetomidine provided a smooth and prolonged post-operative analgesia as compared to clonidine. The evidence was very much visible in the prolonged time to two segmental dermatomal regression (136.46 ± 8.12 min) as well as return of motor power to Bromage 1 (246.72 ± 30.46 min). As a result the time for rescue analgesia was comparatively shorter (310.76 ± 23.75 min) in the patients who were administered clonidine (*P* < 0.05). The superior block characteristics by the addition of dexmedetomidine were clearly evident from the lesser dose consumption (68.54 ± 16.84mg) of ropivacaine for post-operative analgesia for the next 24 hours (*P* < 0.05).

**Table 4:** Comparison of post-op block characteristics in both the groups

Post-op block characteristics (in minutes)	Group RD (n = 25)	Group RC (n = 25)	*P*
Mean time to two segmental regression	136.46 ± 8.12	128.08 ± 7.54	<0.05
Mean time for regression to bromage 1	246.72 ± 30.46	228.44 ± 27.18	<0.05
Mean time to sensory regression at S1	316.64 ± 40.36	296.72 ± 35.52	<0.05
Time to first rescue top-up	342.88 ± 29.16	310.76 ± 23.76	<0.05
Total dose of ropivacaine used (mg)	68.64 ± 17.42	82.52 ± 20.82	<0.05

*P<0.05-S, RC - Ropivacaine + clonidine, RD - Ropivacaine + dexmedetomidine

Table 5 shows the comparative incidence of various side effects in both the groups which were observed in the intra-op and post-op period. The incidence of dry mouth was significantly higher in both the groups but it was statistically non-significant on comparison (*P* > 0.05). The incidence of other side effects like nausea, vomiting, headache, shivering and dizziness were comparable in both the groups and statistically non-significant. We did not observe the respiratory depression in any patient from either group.

**Table 5:** Comparison of side effects observed in both the groups during and after the operative period

Side effects	Group D (n = 25)	Group C (n = 25)
Nausea	4 (16)	3 (12)
Vomiting	1 (4)	1 (4)
Shivering	1 (4)	2 (8)
Headache	1 (4)	1 (4)
Dizziness	3 (12)	2 (8)
Dry mouth	6 (24)	7 (28)
Respiratory depression	0	0

*P<0.05, Figures in parentheses are in percentage

## DISCUSSION

The use of neuraxial opioids is associated with quite a few side effects, so various options including α-2 agonists are being extensively evaluated as an alternative with emphasis on opioid-related side effects such as respiratory depression, nausea, urinary retention and pruritis.[27–29] The pharmacologic properties of α-2 agonists have been extensively studied and have been employed clinically to achieve the desired effects in regional anaesthesia.[8,9,12,13] Epidural administration of these drugs is associated with sedation, analgesia, anxiolysis, hypnosis and sympatholysis.[10,11] Clonidine has been used successfully over the last decade for the said purpose and the introduction of dexmedetomidine has further widened the scope of α-2 agonists in regional anaesthesia.[30,31] The faster onset of action of local anaesthetics, rapid establishment of both sensory and motor blockade, prolonged duration of analgesia into the post-operative period, dose-sparing action of local anaesthetics and stable cardiovascular parameters makes these agents a very effective adjuvant in regional anaesthesia.[32–36]

The present study was undertaken to compare the analgesic efficacy, peri-operative and post-operative, as well as sedation effects of α-2 agonists. The demographic profile of our patients was comparable with respect to mean age, body weight, body mass index, ASA grade and duration of surgery. The results of the study has shown that the addition of either 1.5 μg/kg dexmedetomidine or 2 μg/kg clonidine as adjuvant to epidural ropivacaine not only prolongs the duration of analgesia but also provides a good sedation level during the surgical procedure. Dexmedetomidine has a visible edge over clonidine as it enables an earlier onset and establishment of sensory and motor block. Further, addition of these two adjuvants promotes faster onset compared to established time of onset of sensory analgesia with ropivacaine alone.[37,38]

The results of our study clearly indicate the effectiveness of epidural dexmedetomidine as it produced profound sedation in 36% of the patients, who were arousable by gentle tactile stimulation compared to achievement of similar sedation level in just 16% of the patients in clonidine group. Thirty-two percent of the patients remained awake but calm in clonidine group compared to 16% in dexmedetomidine group who were equally cooperative and calm. Overall, the sedation scores were highly significant statistically with administration of dexmedetomidine.

**Figure 1:**
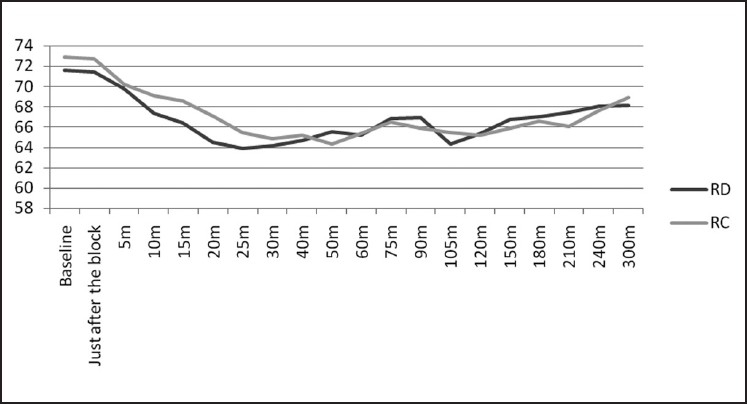
Comparison of heart rate in the group RD and RC covering the pre-op, intra-op and post-operative period

**Figure 2:**
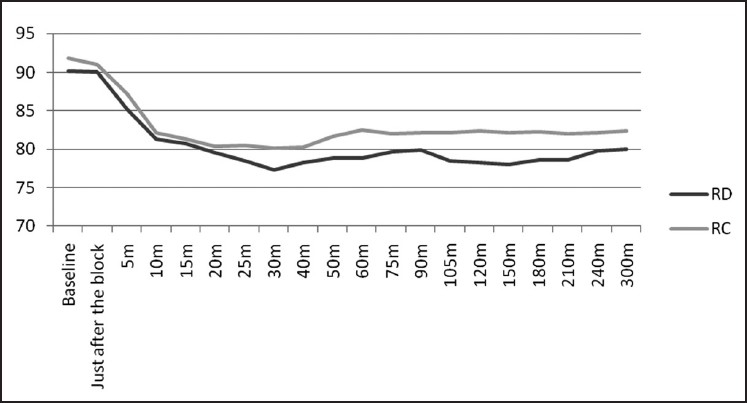
Comparison of mean arterial pressure in the group RD and RC covering the pre-op, intra-op and post-operative period

The RD group showed visible superiority over RC group in various post-operative block characteristics like the weaning of sensory and motor block, prolonged post-operative analgesia and a lesser amount of total ropivacaine used post-operatively. The cardio-respiratory parameters, as is evident from Figures 1 and 2, remained stable throughout the study period which reaffirms the established effects of α-2 agonists in providing a haemodynamically stable peri-operative and post-operative period.[25,39] Although a slight decrease in heart rate and mean arterial pressure was observed in both the groups, it never fell down to more than 15% of the baseline values. The side effect profile of both these drugs was quite favourable as none of the patient in either group had profound deep sedation or respiratory depression which correlates very well with other studies.[20,40,41] Although we observed a little higher incidence of dry mouth and nausea in both the groups, it was only mildly discomforting to the patients and was mainly observed in the post-operative period and non-significant on statistical comparison.

## CONCLUSIONS

We conclude that dexmedetomidine is a better adjuvant than clonidine in epidural anaesthesia as far as patient comfort, stable cardio-respiratory parameters, intra-operative and post-operative analgesia is concerned. Overall the experience with dexmedetomidine was quite satisfactory as compared to clonidine because of its superior sedative and anxiolytic properties during the surgical procedure under regional anaesthesia.
